# Pyogenic vertebral osteomyelitis of the elderly: Characteristics and outcomes

**DOI:** 10.1371/journal.pone.0188470

**Published:** 2017-12-05

**Authors:** Johan Courjon, Adrien Lemaignen, Idir Ghout, Audrey Therby, Nadia Belmatoug, Aurélien Dinh, Guillaume Gras, Louis Bernard, on behalf of the DTS (Duration of Treatment for Spondylodiscitis) study group

**Affiliations:** 1 Department of Infectious Diseases, University Hospital of Nice, Hôpital Archet 1, Nice, France; 2 Université Côte d’Azur, Nice, France; 3 Department of Internal Medicine and Infectious Diseases, University Hospital of Tours, Hôpital Bretonneau, Tours, France; 4 François Rabelais University, Tours, France; 5 Clinical Research Unit, Assistance Publique Hôpitaux de Paris, Hôpital Ambroise Paré, Boulogne, France; 6 Department of Internal Medicine and Infectious Diseases, Centre Hospitalier de Versailles, Hôpital Mignot, Le Chesnay, France; 7 Department of Internal Medicine, Assistance Publique Hôpitaux de Paris, Hôpital Beaujon, Clichy, France; 8 Department of Infectious Diseases, Assistance Publique Hôpitaux de Paris, Hôpital Raymond Poincaré, Garches, France; Aga Khan University - Kenya, KENYA

## Abstract

**Background:**

The incidence of pyogenic vertebral osteomyelitis (PVO) has increased over the past two decades. One possible cause of this increase is the aging of the population, which results in more comorbidities in high income countries.

**Objective:**

To better characterize the clinical presentation and outcome of PVO in the elderly.

**Design:**

We conducted a post-hoc analysis of a previously published trial that studied treatment duration in PVO and compared the presentation and outcomes according to age.

**Participants:**

Our analysis included 351 patients among whom 85 (24%) were 75-years-old or more.

**Results:**

There were no significant differences in the socio-demographics of the patients. Neoplasia and chronic inflammatory diseases were more common in the older group: 34% vs. 19% (p = 0.021) and 9% versus 1% (p = 0.004), respectively. There were no significant differences in clinical and radiological presentations between the groups in terms of back pain (337/351, 97%), fever (182/351, 52%), PVO localization, neurological signs and epidural abscess. Associated infective endocarditis (IE) was more frequent in the older group (37% vs. 14%, p<0.001). Streptococci were more frequently involved in infections of older patients (29% vs. 14%, p = 0.003) in contrast to Staphylococcus aureus (31% vs. 45%, p = 0.03). Older patients displayed higher mortality rates at 1 year (21% vs. 3%, p<0.001) and more adverse events related to cardiorespiratory failure (10.6% vs. 3.8%, p = 0.025), but had similar quality of life among the survivors.

**Conclusion:**

During PVO, the clinical and radiological findings are similar in older patients. Global mortality rates are higher in older patients compared to younger patients, which could be explained by the increased frequency of neoplasia at diagnosis and higher prevalence of associated IE in the elderly.

## Introduction

Pyogenic vertebral osteomyelitis (PVO) is a rare condition with an estimated annual incidence in France of 2.4/100 000 inhabitants. In patients aged 70 years or more, the incidence reached 6.5/100 000 inhabitants in 2002–2003 [1]. A recent Spanish study that analyzed the trends in bone and joint infections reported an increasing incidence of PVO in older patients (≥ 65 years) compared to younger patients [≤ 49 years over a 26 year-period (1985–2011)] [2]. Diagnosis can be difficult due to the lack of specific clinical signs and variable febrile symptoms. Back pain has been reported in 86% of PVO cases [3], but such a complaint is common and is made by up to 40% of females between 60 and 69 years of age [4]. Studies focusing on PVO in elderly cohorts are scarce and often rely on case series. The impact of age on the outcomes of PVO has differed across many studies.

In an open-label, randomized clinical trial, we demonstrated that 6 weeks of antibiotics were non-inferior to 12 weeks of treatment for patients presenting with PVO [5]. Among the 351 patients included, 85 (24%) were older than 75 years, which as one of the factors associated with treatment failure in the multivariable analysis.

In this case-control ancillary study, we aimed to describe the clinical characteristics, radiological findings, microbiological epidemiology and outcomes of PVO in this specific population of elderly patients.

## Patients and methods

This post-hoc analysis was conducted using patient data extracted from the previously published multi-centric randomized controlled trial (RCT) NCT00764114, which investigated the optimal duration of antibiotics for the treatment of PVO [5]. Overall, 359 patients with PVO underwent randomization for 6 or 12 weeks of antibiotics treatment between November 15, 2006, and March 15, 2011, in 71 medical centers in France. To be included, patients had to be adults with typical radiological findings of PVO associated with microbiological documentation, a life expectancy longer than one year, and no device at the site of the infection.

The main objective of this case-control study was to describe demographic and symptom data from a large series of aged patients suffering from PVO and to compare these findings to a younger population. The secondary objective was to determine the link between age and outcome.

Patients included in the intention-to-treat (ITT) analysis of the RCT were therefore divided into two groups according to the age at diagnosis. Given the lack of a consensus definition, elderly patients were defined arbitrarily as patients aged 75 years or more. We used variables recorded in the RCT as previously described.

The comparison between the groups was performed using a univariate analysis that employed the appropriate statistical parameters according to the nature of the variables. Distributions were compared using the Chi-square test or Fisher’s exact test if necessary. Continuous quantitative variables and ordinal variables were tested using the non-parametric method of Kruskal-Wallis.

In the RCT, 160 (90.9%) of 176 patients in the 6-week group and 159 (90.9%) of those in the 12-week group met the criteria for clinical cure. The factors associated with unfavorable outcomes were determinate by a multivariable logistic regression analysis. The explanatory variables included in the model were those that had a degree of significance (p) below 5% in the univariate analysis: age (≥75 years or <75 years), infective endocarditis (IE) (yes or no), *Staphylococcus aureus* infection (yes or no), positive blood culture (yes or no), treatment with oral fluoroquinolone and rifampicin (yes or no), duration of intravenous antibiotic treatment (<7 days or >7 days), and allocated duration of treatment (6 weeks or 12 weeks). We examined the effects of variable exclusion on the Akaike information criterion (AIC) and chose the model with the smallest AIC. All statistical analyses were performed using R software version 3.1 [6].

The French National Agency for the Safety of Medicines and Health Products, French Data Protection Agency, and ethics committee of the Versailles University Hospital approved the study protocol. The study was performed in accordance with the ethical principles of the Declaration of Helsinki and the guidelines for Good Clinical Practice. Written informed consent for participation in the trial was obtained from all patients.

## Results

**Fig 1:**
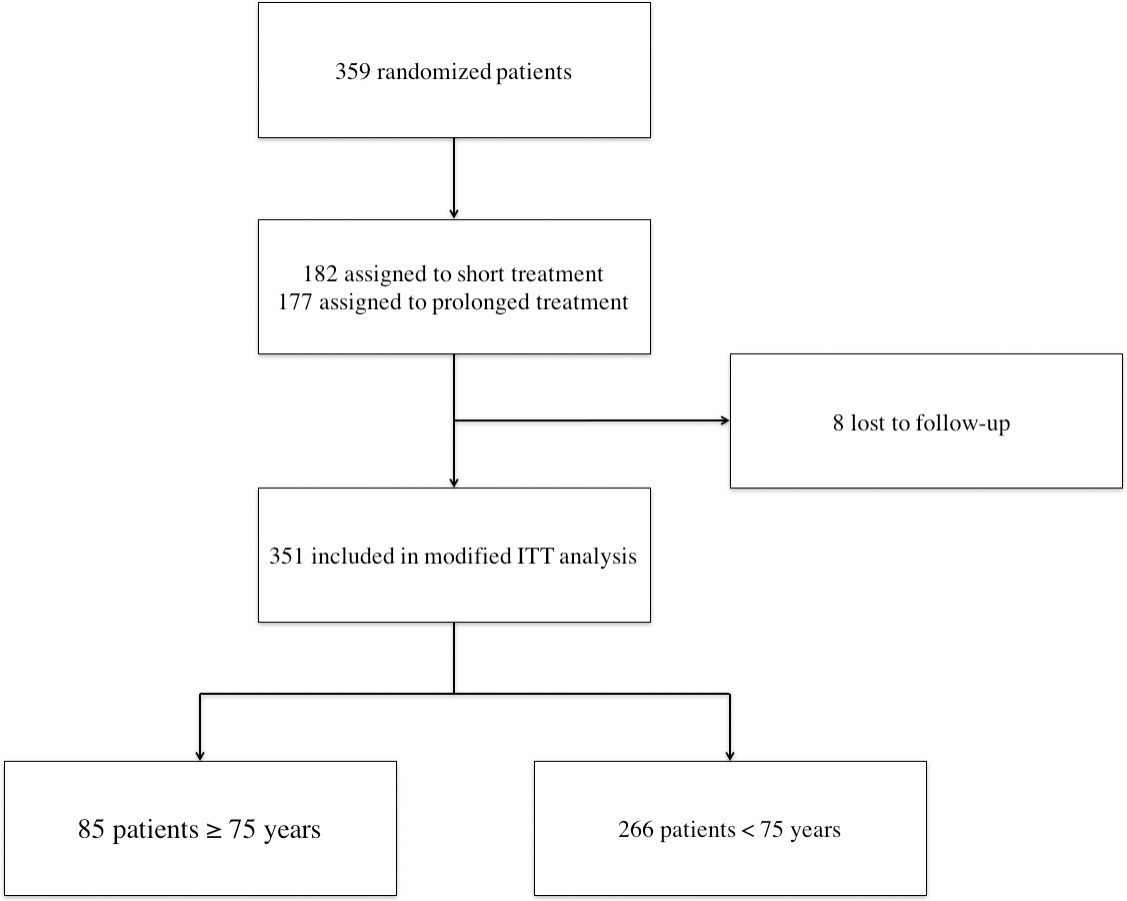
Flow-chart. All patients were included in the Duration of treatment for Spondylodiscitis study. Short treatment: 6 weeks of antibiotic treatment. Prolonged treatment: 12 weeks of antibiotic treatment. ITT: intention to treat.

Among the 351 patients included in the ITT analysis of the RCT, 85 (24%) were 75 years or older (mean age: 80.5 ± 4.2 years) and 266 (76%) were younger than 75 years of age (mean age: 55 ± 14.1 years; Fig 1). The comorbidities of patients in both groups are presented in Table 1. Significantly more patients had neoplasia and chronic inflammatory disease in the ≥75-year-old group. The duration between the onset of symptoms and diagnosis was similar (45.3 ± 43 days *vs* 49.9 ± 59.1 days, p = 0.929). Back pain and fever at diagnosis were present in 337 patients (96%) and 182 patients (52%), respectively, and did not significantly differ between the two groups. One week after enrollment, fever persisted more often in older patients (13.2% *vs*. 3%, *p* = 0.004), and this difference disappeared after further follow-up observations. The presence of neurological symptoms and the localization of the PVO were similar in both age groups. The main features of PVO presentation in our patients are shown in Table 2.

**Table 1:** Description of the study population.

	≥ 75 years (n = 85)	< 75 years (n = 266)	*p*
Women	37 (43.5)	72 (27.1)	**0.007**
Malnutrition	9 (13)	11 (5.6)	0.083
Diabetes mellitus	13 (18.6)	41 (20)	0.932
Alcohol abuse	1 (1.5)	30 (14.8)	**0.007**
Smokers	11 (16.2)	108 (48.2)	**<0.001**
Cirrhosis	0 (0)	8 (4.1)	0.206
Neoplasm	22 (33.8)	38 (19)	**0.021**
Central venous catheter	3 (4.4)	13 (6.8)	0.769
Renal insufficiency	1 (1.6)	4 (2.1)	1
Respiratory insufficiency	2 (3.1)	7 (3.5)	1
Immunocompromised host	5 (7.4)	11 (5.6)	0.567
Chronic inflammatory disease	6 (9.1)	2(1)	**0.004**
Randomized in 12-weeks arm	40 (47.1)	135 (50.8)	0.640

Numbers in brackets are the percentages of the sub-group populations. Bolded p-values are considered to be significant as they are lower than 0.05.

**Table 2:** Clinical and radiological presentation of vertebral osteomyelitis.

	≥ 75 years (n = 85)	< 75 years (n = 266)	*p*
Fever	49 (57.6)	133 (50.2)	0.283
Back pain	80 (94.1)	257 (96.6)	0.34
Neurological symptoms			
Radiculopathy	6 (7.1)	33 (12.4)	0.243
Spinal cord compression	3 (3.5)	16 (6)	0.582
PVO localization			
Multiple	12 (14.1)	26 (9.8)	0.357
Cervical	9 (10.6)	43 (16.2)	0.278
Thoracic	28 (32.9)	69 (25.6)	0.984
Lumbar	59 (69.4)	187 (70.3)	0.086
Sacral	6 (7.1)	39 (14.7)	0.101
Epidural Inflammation	20 (24.7)	91 (35.8)	0.567
Infective endocarditis	25 (36.8)	26 (13.8)	**<0.001**

Numbers in brackets are the percentages of the sub-group populations. Bolded p-values are considered to be significant as they are lower than 0.05. PVO: pyogenic vertebral osteomyelitis. Localization of PVO describes the spinal level affected; the sum of the values exceeds the number of total cases because of the presence of PVO in multiple localizations.

The diagnosis of IE was assessed by echocardiography in 257 (73.2%) patients (transesophageal echocardiography for 227 patients). Bacteremia was more frequent in the ≥75-year-old group (83.5% *vs*. 63.5%, *p*<0.001). In the older group, 25 patients (36.8%) met the Duke criteria for IE compared to 26 (13.8%) in the <75-year-old group (*p*<0.001).

Microbiological diagnosis of PVO was performed using blood cultures for 240 (68%) patients, CT-guided vertebral biopsy for 138 patients (39%) and perioperative surgical biopsy for 19 patients (5%). While *Staphylococcus aureus*, regardless of the susceptibility test results, was more frequent in the <75-year-old group (30.6% *vs*. 44.7%, *p* = 0.029), methicillin-resistant *S*. *aureus* (MRSA) was involved in 8/351 patients (2.3%) and more frequently in the ≥75-year-old group (33.3% *vs*. 5.6%, *p* = 0.013). *Streptococcus* spp. were also more likely to be isolated from older patients (29.4% *vs*. 14.3%, *p* = 0.003); the microbiological identification results did not show any significant differences for coagulase-negative *Staphylococci* (17.6% *vs*. 17.3% *p* = 1), *Enterococcus* spp (12.9% *vs*. 10.2% p = 0.603) or *Enterobacteriaceae* (3.5% *vs*. 3.8% *p* = 1).

A higher rate of severe adverse events was recorded in the ≥75-year-old group (45.9% *vs*. 23.3%, *p*<0.001), particularly with respect to cardiorespiratory failure (10.6% *vs*. 3.8%, *p* = 0.025). There were no significant differences in adverse events due to antimicrobials, *Clostridium difficile* associated diarrhea (1.2% *vs*. 1.1%, *p* = 1) or neurological complications (10.6% *vs*. 18%, *p* = 0.146) of the PVO. The median hospital length of stay was significantly longer in the elderly cohort, with durations of 23 days (Inter Quartile Range: IQR = 15) and 26 days (IQR = 13) for the younger and older groups (p = 0.02), respectively. Regardless of the duration of antibiotic treatment (6 or 12 weeks), age ≥ 75 years was associated with treatment failure according to a multivariable analysis (OR [95% CI]: 1.08 [1.01–1.16], p = 0.028; please see S1 Table for the other risk factors involved in failure of treatment). Death occurred in 18 (21.2%) patients in the elderly group versus 8 (3%) in the younger group (*p*<0.001). Regarding quality of life during the follow-up, the EQ-5D score was significantly lower in the ≥75-year-old group after 6 months (0.5 ± 0.3 *vs*. 0.7 ± 0.4, *p =* 0.007), but there was no significant differences observed at the one year follow-up.

## Discussion

This post-hoc analysis of the DTS study provides interesting results and enables a better understanding of PVO occurring in elderly patients. The main strengths of this study are the sample size and homogeneity of data collected prospectively through the RCT. To our knowledge, this is the largest representative cohort of aged patients with PVO. The description of specific aspects due to advanced age is of the highest importance in these times of an increasing incidence of PVO [2,7,8] and aging population. The main limits of this study are the post-hoc characteristics of the analysis and its primary descriptive angle.

The World Health Organization’s definition of “aged” is someone who is older than 65 years of age, but this is probably not fully applicable to high income countries where aging populations represent a high proportion of residents. Of note, 65 years is also the median age of patients presenting with PVO in France, and therefore, this definition does not represent a discriminant cut-off. The choice of 75 years of age as a cut-off is somewhat arbitrary but is often chosen in daily practice, including for administrative permission for admission into retirement homes. This cut-off was chosen in the multivariable regression logistic model developed in the DTS study to identify the predictive factors of failure. Not surprisingly, the sex ratio was in favor of females in older patients suffering from PVO in the same way as the whole population of this age. Clinical symptoms did not differ significantly from younger patients, especially with respect to fever, which was detected in the same proportion of younger and older patients. However, older patients displayed poorer outcomes with higher mortality rates and more adverse events, which were not explained by a delay in management initiation or an increase in PVO-related severity of neurological complications. Neoplasia and chronic inflammatory diseases were the two conditions in the ≥75-year-old group that significantly differed from younger patients.

The microbiological results and IE diagnosis might offer some interesting hypotheses to explain these discrepancies in outcome. There are contradictory data on the influence of age on PVO outcomes. One series included patients aged from 60 to 84 years who required neurosurgical management with no lethality recorded [9]. Others reported that being under the age of 60 was an independent factor predicting favorable outcome in non-surgical patients [10]. The higher mortality rate recorded in our study for older patients is in accordance with pervious published results. In a Japanese retrospective observational study including 7118 PVO patients, in-hospital mortality increased proportionally with age and was significantly influenced by IE co-infection [11]. In the study of McHenry *et al*. [12] reporting the long-term outcomes of 253 patients with PVO with a 6.5 year median duration follow-up, the mortality rate was 11%. The authors compared patients under or over 50 years and did not find any differences in terms of outcome. The association with IE was not studied. The cut-off age of 50 years and fact that most of the patients underwent surgical management do not allow for a direct comparison with our study.

Unlike others, we did not find an increase in the proportion of PVO cases that were caused by Gram-negative bacilli in our aged population. It should be noted that the first reports on the abundance of such bacteria were case series or retrospective works [13–15] and, as such, they do not appear to be representative of larger populations.

Patients in the ≥75-year-old group presented more frequently with *Streptococcus* spp PVO and IE. If the pathophysiological determinants of those results in this specific age group remain unclear, the association between *viridans Streptococci* species and IE during PVO has already been reported to be significantly stronger than with *S*. *aureus* [16]. Two other studies reported an association between PVO caused by *Streptococcus* spp or *Enterococcus* spp and the diagnosis of associated IE [17,18]. Similarly, a retrospective series of 92 IE patients identified 14 with PVO. *Streptococcus* spp were involved in 8 of these cases compared to only one with a *S*. *aureus* infection [19]. The increased incidence of *Viridans Streptococci* and *Streptococcus gallolyticus* infections during IE in elderly compared to younger patients (<65 years) has already been reported [20]. However, cases of IE caused by *Viridans Streptococci* are associated with a decrease in in-hospital mortality rates compared to IE caused by other bacteria [21]. Thus, it is likely that the higher incidence of IE contributes to the unfavorable outcomes.

## Conclusion

While PVO diagnosis is not easy and requires attentive physicians, it does not seem to be more difficult in the elderly. The initial presentation does not differ significantly in terms of symptoms or severity. The microbiological findings are different, with fewer *Staphylococci* infections but a higher prevalence of MRSA and *Streptococci* spp infections in the elderly. The global mortality rate at one year is higher in patients older than 75 years of age and could be explained by a higher frequency of associated IE and higher rate of comorbid conditions, such as neoplasia. In this population, looking for IE should be mandatory, especially when *Streptococci* spp are involved.

## Supporting information

S1 Table Risk factors for failure of treatment in PVO. The Akaike Information Criterion (AIC) of the final model was 108.9. OR = Odds ratio. aOR = adjusted Odds ratio. CI = confidence interval.
(DOCX)
