# Supplementary material for: Pyogenic vertebral osteomyelitis of the elderly: Characteristics and outcomes
Source: PLoS One. 2017 Dec 5;12(12):e0188470. doi: 10.1371/journal.pone.0188470 (PMC5716588; doi:10.1371/journal.pone.0188470)
Supplement: S1 Table — The Akaike Information Criterion (AIC) of the final model was 108.9. OR = Odds ratio. aOR = adjusted Odds ratio. CI = confidence interval. (DOCX) [file pone.0188470.s001.docx]

**S1 Table. Risk factors for failure of treatment in PVO**

|  | **Failure** | **Univariate analysis** | | **Multivariable analysis** | |
| --- | --- | --- | --- | --- | --- |
|  |  | **OR [95% CI]** | **p** | **aOR [95% CI]** | **p** |
| **Age** |  |  |  |  |  |
| >=75 | 13/85 (15.3) | 1.08 [1.01 - 1.16] | 0.023 | 1.08 [1.01 - 1.16] | 0.028 |
| <75 | 19/266 (7.1) |  |  |  |  |
| ***S. aureus* infection** |  |  |  |  |  |
| Yes | 22/145 (15.2) | 1.11 [1.04 - 1.18] | 0.001 | 1.16 [1.08 - 1.24] | <0.001 |
| No | 10/206 (4.9) |  |  |  |  |
| **Endocarditis** |  |  |  |  |  |
| Yes | 9/56 (16) | 1.09 [1 - 1.18] | 0.049 | 1.08 [0.99 - 1.18] | 0.074 |
| No | 23/295 (7.8) |  |  |  |  |
| **Fluoroquinolone or**  **rifampin treatment** |  |  |  |  |  |
| Yes | 14/155 (9) | 1 [0.94 - 1.06] | 0.961 | 0.95 [0.88 – 1.03] | 0.113 |
| No | 18/196 (9.2) |  |  |  |  |

The Akaike Information Criterion (AIC) of the final model was 108.9. OR=Odds ratio. aOR=adjusted Odds ratio. CI= confidence interval.
